# Association of Hypocapnia With Febrile Seizures: A Cross-Sectional Observational Study

**DOI:** 10.7759/cureus.77376

**Published:** 2025-01-13

**Authors:** Archana S, Karunya Ravi, Prasanna Raju, Suresh Rangaraj

**Affiliations:** 1 Department of Paediatrics, Sri Ramaswamy Memorial (SRM) Medical College Hospital and Research Centre, Chennai, IND

**Keywords:** alkalosis, febrile seizures, hypocapnia, neurology, respiratory alkalosis

## Abstract

Background: Febrile seizures are a benign condition with unexplained pathophysiology. Respiratory alkalosis due to increased temperature can cause cerebral alkalosis and seizures.

Objectives: The objective of this study is to assess the association of hypocapnia and respiratory alkalosis with the time of presentation after febrile seizures.

Study design: This is a cross-sectional observational study.

Participants: A total of 51 children admitted to SRM Medical College Hospital over a period of one year (September 2020-September 2021) were included in the study.

Intervention: Venous blood gas was measured at admission. The presence of hypocapnia and respiratory alkalosis were analyzed along with the time of admission from the onset of seizures.

Results: When comparing temperature values with pH values as a linear regression, there was a positive correlation (r = 0.39, P = 0.005). Children who presented earlier were more likely to have respiratory alkalosis compared to those who presented late (P = 0.000).

Conclusions: Febrile seizures could be associated with blood pH changes, making way for newer therapeutic approaches and the need for subsequent clinical trials.

## Introduction

Febrile seizures, even though they are the most common convulsive event in children, largely remain unexplained. They are febrile (temperature ≥ 100.4° F or 38° C by any method) convulsive episodes that occur in the absence of a central nervous system (CNS) infection in children between 6 and 60 months of age [1]. The incidence of febrile seizures is around 2-5% of all children younger than 60 months old [2]. Febrile seizures are generally considered benign, but studies have shown their association with long-term complications such as temporal lobe epilepsy and intellectual disability in children with pre-existing neurological illnesses [3,4]. Recurrence of febrile seizures is observed in one-third of the children [1], and based on its characteristics, it can be classified as simple or complex. A simple febrile seizure is a generalized seizure that lasts for less than 15 minutes and does not recur within 24 hours. A focal prolonged seizure lasting more than 15 minutes, recurring within 24 hours is a complex febrile seizure [5]. Although febrile seizures have been attributed to many genetic and inflammatory factors, the exact pathophysiology remains unexplained [6,7].

A rise in body temperature is associated with an increase in an individual’s respiratory rate [8]. This hyperthermia-induced hyperventilation can alter blood gases and pH by causing carbon dioxide washout and subsequent respiratory alkalosis. Alkalosis thus formed could be the underlying reason for neuronal hyperexcitability, exhibited as febrile seizures [9]. This mechanism of febrile seizures was demonstrated using animal models [10,11].

The objectives of this study were to assess the association of hypocapnia and respiratory alkalosis with the time of presentation after febrile seizures and to assess the correlation between hypocapnia, respiratory alkalosis, and various clinical and demographic variables. 

## Materials and methods

This was a cross-sectional observational study conducted at SRM Medical College and Hospital, Kattankulathur, Chennai, and approved by the Institutional Ethics Committee. During the period between September 2020 and September 2021, 51 children were enrolled in the study after obtaining written informed consent from their parents. 

This study included children between the ages of six months and six years who presented to the Emergency Department with an axillary temperature of more than 37.8° C and seizures with no previous neurological disorders, as well as developmentally normal children. Children who presented more than 24 hours after febrile seizures, children suspected to have meningitis, and children with complex febrile seizures were excluded from the study. Children with lower respiratory tract infections and respiratory distress were also excluded from the study, as respiratory distress might alter blood gasses. The sample size was calculated using the formula 4*P*Q/ d2, with an allowable error of 7.

**Figure 1:**
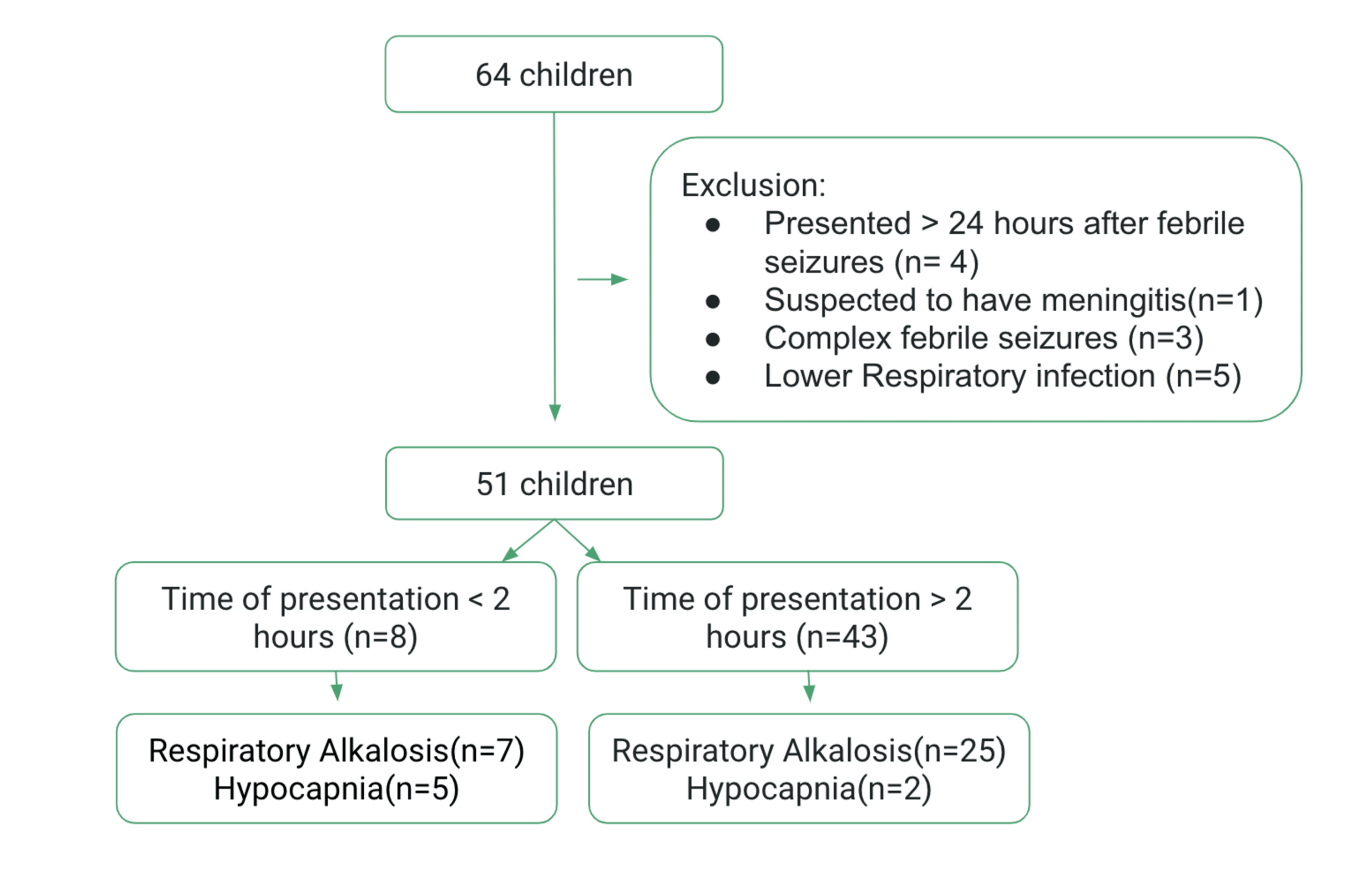
Process flow diagram

Venous blood samples were taken soon after patients presented to casualty, under aseptic precautions using heparinized syringes. Blood gas analysis was done using the ABL800 FLEX Potentiometric Measuring Principle and compared with the standard values pH 7.35-7.45; pCO2 35-45 mm Hg. A digital thermometer was used to check patients’ axillary temperature twice, and the mean of the two readings was taken. The patient’s demographic details were documented, and a thorough neurological examination was conducted, including analyses of higher mental function, motor and sensory systems, cranial nerves, cerebellar function, and the autonomic nervous system. Details such as time of presentation, type of seizure, age, and family history of febrile seizures were also noted, and capillary blood sugar was checked. Based on the time of presentation to the hospital after the seizure episode, these children were divided into two groups: (a) children who presented within two hours and (b) children who presented more than two hours later (Figure 1).

Categorical and nominal variables were analyzed using a chi-squared test, and a Student’s t-test was used for the continuous variables. We analyzed correlation and linear regression between pH and pCO2 with temperature.

## Results

Of the 51 children enrolled in the study, the majority were in the age group of 12-24 months (70.6%; n = 36), with a mean of 20.78 +/- 7.88 SD. Of the children, 84% (n = 43) reported to the hospital after the first two hours after a febrile seizure, and 58% (n = 30) of them had a positive family history of febrile seizures. At the time of presentation, 96% (n = 49) of the patients had fever. Out of the total population, 25% (n = 13) had an abnormal pulse rate (age appropriate as per PALS guidelines), and around 6% (n = 3) had an abnormal respiratory rate (age appropriate as per PALS guidelines). The majority of the children presented with generalized seizures (92%; n = 47) (Table 1). 

**Table 1:** Hypocapnia and respiratory alkalosis in association with other variables

Variables	Hypocapnia	Respiratory alkalosis
Time of presentation		
Within two hours	78% (n= 25)	4.66% (n=2)
More than two hours	21% (n= 7)	62.5% (n=5)
	(P= 0.231)	(P=0.000)
Type of seizures		
Generalised	93.8% (n=30)	100% (n=7)
Undefined	6.3% (n=2)	0
	(P= 0.623)	(P=0.629)
Age		
< 1 year	10.5% (n=2)	28.6% (n=2)
1-2 years	68.4% (n=23)	42.9% (n=3)
2-3 years	15.8% (n=6)	14.3% (n=1)
3-4 years	5.3% (n=1)	14.3% (n=1)
	(P=1.000)	(P=0.058)
Family history		
Present	50% (n=16)	28.6% (n=2)
Absent	50% (n=16)	71.4% (n=5)
	(P=0.143)	(P=0.109)
Presence of fever		
Present	96.9% (n= 31)	100% (n=7)
Absent	3.1% (n= 1)	0
	(P= 1.000)	(P=1.000)
Abnormal pulse rate		
Normal	65.6% (n=21)	100% (n=7)
Abnormal	34.4% (n=11)	0
	(P= 0.96)	(P=0.169)
Abnormal respiratory rate		
Normal	96.9% (n=31)	100% (n=7)
abnormal	3.1% (n=1)	0
	(P=0.547)	(P=1.000)
Capillary blood glucose		
Normal	87.5% (n=28)	100% (n=7)
Abnormal	12.5% (n=4)	0
	(P=0.283)	(P=0.629)

Venous blood gas analysis done at the time of admission revealed that 62.7% (n = 32) of the study population had hypocapnia (pCO2 < 35mm Hg), but only 14% (n = 7) of the children had respiratory alkalosis. Among the children who had hypocapnia, about 21.9% (n = 7) of them presented to the hospital within two hours of seizure onset, and the remaining 78.1% (n = 25) presented to the hospital more than two hours after seizure onset (P = 0.231).

Respiratory alkalosis was more frequently observed in children who presented within the first two hours of seizure onset (62.5%; n = 5) when compared to children who presented late (4.66%; n = 2). The association was found to be statistically significant (P = 0.000).

**Figure 2:**
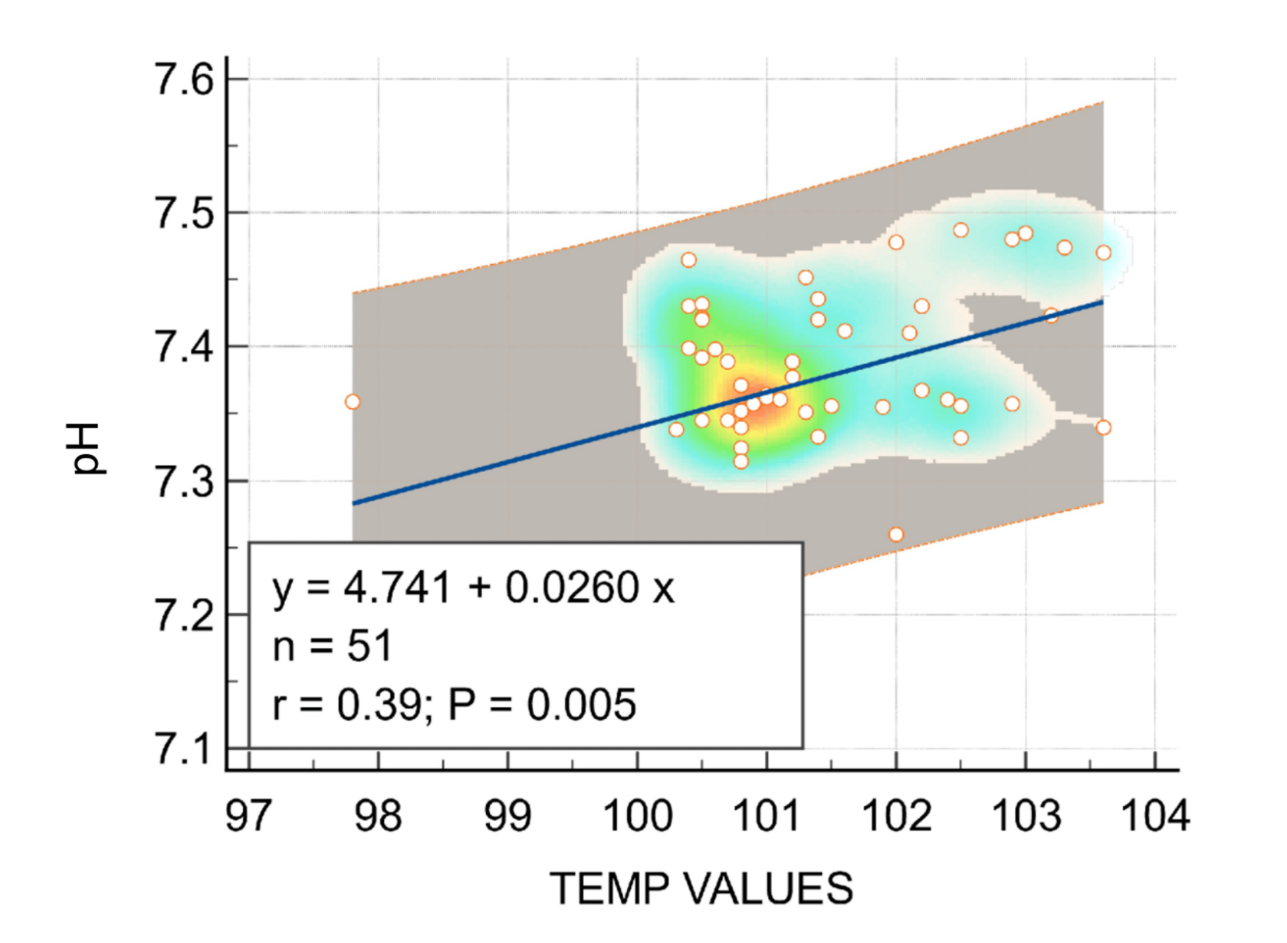
Correlation between temperature and pH

When the temperature value in Fahrenheit was compared with the pH value as a linear regression, there was a positive correlation between temperature and pH (r = 0.39, P = 0.005; Figure 2).

**Figure 3:**
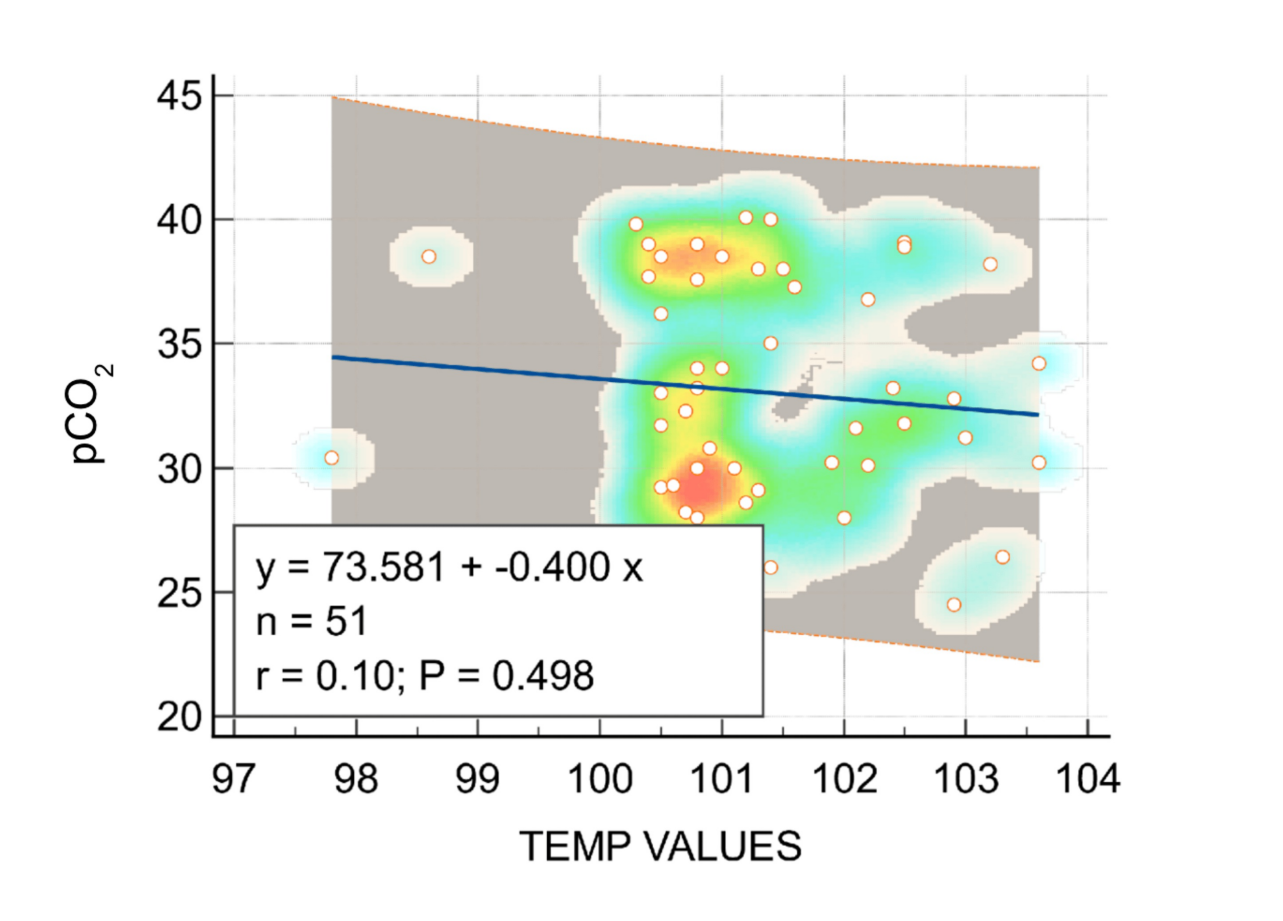
Correlation between temperature and pCO2

However, when the association between temperature values and pCO2 was studied, there was no significant correlation between temperature and pCO2 (r0.097, P = 0.498; Figure 3).

## Discussion

The study focused on examining the relationship between febrile seizures and blood pH changes. Animal models of alkalosis-induced seizures were demonstrated in a study by Schuchmann et al. [10]. Experimental febrile seizures were induced in lab rats, showing that a rise in brain pH can enhance neuronal excitability, with a threshold of 0.2-0.3 pH units for seizure induction. They also demonstrated that suppressing alkalosis with 5% ambient CO2 abolished seizures within 20 minutes, suggesting a possible therapeutic approach. The study has also shown that suppressing alkalosis is associated with decreased long-term complications such as the upregulation of the Ih current in the hippocampus and the upregulation of the CB1 receptor expression. Morimoto et al. showed that seizure duration was longer at lower pCO2 [12].

In our study, we found that while 32 children (62.7%) had hypocapnia after admission, 19 children did not have hypocapnia. The reason for the presence of normal pCO2 levels in the remaining 37.3% following febrile seizures could be linked to a delay in bringing the child to the hospital. In a study conducted by Sachan and Goyal [13], 91% of the children had hypocapnia. We were unable to show a significant correlation between hypocapnia in children presented before and after two hours post-seizure. However, Sachan and Goyal showed a positive correlation between hypocapnia and time of seizures. This could be attributed to the reason being a higher number of children (91%) presenting with hypocapnia in their study.

In our study, seven children (13.7%) had respiratory alkalosis, and 44 children did not. Similarly, Sachan and Goyal observed respiratory alkalosis in 20% of the children studied [13]. The disparity in the percentage of hypocapnia and respiratory alkalosis in both studies can be attributed to the delay in bringing the child to the hospital. The fact that the pH will normalize earlier than pCO2 due to the presence of various buffers and hypocapnia may be compensation if there is metabolic acidosis, which was seen in a significant number of children (17.6%) in our study.

To understand the protective role of acidosis in febrile seizures, Schuchmann et al. studied the acid-base levels of children with febrile seizures and gastroenteritis, a condition known to cause acidosis, and they found that febrile seizures were not observed in children with gastroenteritis [14]. 

In another study, Kilicaslan et al. compared the acid-base status of children with a febrile illness not associated with febrile seizures with that of children who presented with febrile seizure, and there was no significant difference [15]. This raises the possibility of genetic predisposition in certain children.

Limitations of our study include the low sample size and the fact that the data collected was in the post-ictal period. The crucial phase of acid-base derangement during or immediately after the ictal phase is difficult to study in any design. The low sample size in our study is due to the COVID-19 pandemic during the study period.

In our study, we compared the acid-base status of children presenting within two hours of the seizure episode and after two hours. Those children who presented early (< two hours) had significantly more respiratory alkalosis when compared to those children who presented late (> two hours). However, there was no positive correlation between respiratory alkalosis and any other variables (e.g., type of seizures, fever at admission, and vital parameters including pulse rate and respiratory rate; Table 1). There was also no positive correlation between respiratory alkalosis and blood glucose at admission. When temperature values were compared with pH values as a linear regression, there was a positive correlation between temperature and pH (r = 0.39; P = 0.005; Figure 2). No significant correlation between temperature and pCO2 could be established (r = -0.097, P = 0.498; Figure 3), which could be a result of some children (n = 9) having metabolic acidosis, and hypocapnia could have been due to compensation.

## Conclusions

Despite being the most common convulsive event among children, the mechanism behind febrile seizures is unexplained, which can cause disagreement in terms of management. We found that as temperature increased in children with febrile seizures, their pH became more alkalotic, which was statistically significant.

Though pCO2 became more hypocapnic, it was not statistically significant. No association could be established between hypocapnia and time of presentation to the hospital, but children who presented early were more likely to have respiratory alkalosis when compared to those children who presented late, which was statistically significant. 
